# Plasma cell dependence on histone/protein deacetylase 11 reveals a therapeutic target in multiple myeloma

**DOI:** 10.1172/jci.insight.151713

**Published:** 2021-12-22

**Authors:** AGM Mostofa, Allison Distler, Mark B. Meads, Eva Sahakian, John J. Powers, Alexandra Achille, David Noyes, Gabriela Wright, Bin Fang, Victoria Izumi, John Koomen, Rupal Rampakrishnan, Tuan P. Nguyen, Gabriel De Avila, Ariosto S. Silva, Praneeth Sudalagunta, Rafael Renatino Canevarolo, Maria D. Coelho Siqueira Silva, Raghunandan Reddy Alugubelli, Hongyue A. Dai, Amit Kulkarni, William S. Dalton, Oliver A. Hampton, Eric A. Welsh, Jamie K. Teer, Alexandre Tungesvik, Kenneth L. Wright, Javier Pinilla-Ibarz, Eduardo M. Sotomayor, Kenneth H. Shain, Jason Brayer

**Affiliations:** 1 Immunology Program,; 2 Department of Malignant Hematology,; 3 Chemical Biology and Molecular Medicine Program,; 4 Proteomics & Metabolomics Core Facility, and; 5 Department of Cancer Physiology, Moffitt Cancer Center & Research Institute, Tampa, Florida, USA.; 6 George Washington University Cancer Center, Washington DC, USA.; 7 M2Gen, Tampa, Florida, USA.; 8 Department of Biostatistics and Bioinformatics, Moffitt Cancer Center & Research Institute, Tampa, Florida, USA.

**Keywords:** Immunology, Oncology, Bone marrow differentiation, Cancer

## Abstract

The clinical utility of histone/protein deacetylase (HDAC) inhibitors in combinatorial regimens with proteasome inhibitors for patients with relapsed and refractory multiple myeloma (MM) is often limited by excessive toxicity due to HDAC inhibitor promiscuity with multiple HDACs. Therefore, more selective inhibition minimizing off-target toxicity may increase the clinical effectiveness of HDAC inhibitors. We demonstrated that plasma cell development and survival are dependent upon HDAC11, suggesting this enzyme is a promising therapeutic target in MM. Mice lacking HDAC11 exhibited markedly decreased plasma cell numbers. Accordingly, in vitro plasma cell differentiation was arrested in B cells lacking functional HDAC11. Mechanistically, we showed that HDAC11 is involved in the deacetylation of IRF4 at lysine^103^. Further, targeting HDAC11 led to IRF4 hyperacetylation, resulting in impaired IRF4 nuclear localization and target promoter binding. Importantly, transient HDAC11 knockdown or treatment with elevenostat, an HDAC11-selective inhibitor, induced cell death in MM cell lines. Elevenostat produced similar anti-MM activity in vivo, improving survival among mice inoculated with 5TGM1 MM cells. Elevenostat demonstrated nanomolar ex vivo activity in 34 MM patient specimens and synergistic activity when combined with bortezomib. Collectively, our data indicated that HDAC11 regulates an essential pathway in plasma cell biology establishing its potential as an emerging theraputic vulnerability in MM.

## Graphical abstract

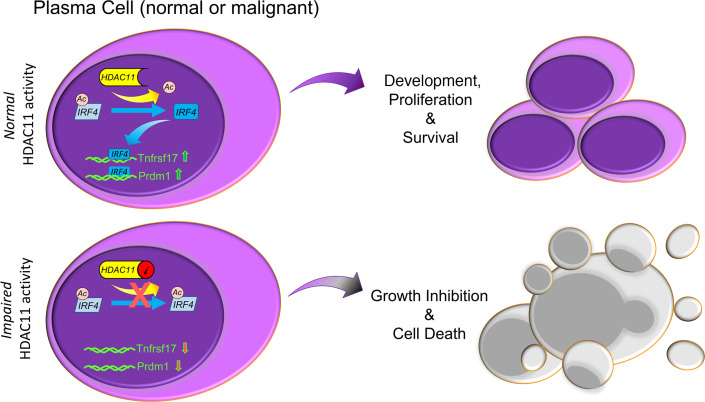

## Introduction

Multiple myeloma (MM) is the second most common hematological malignancy. In 2020, there were an estimated 32,270 new cases and MM caused 12,830 deaths in the United States alone; the overall 5-year life expectancy is only 53.9% (1). The treatment of MM has greatly evolved over the past 2 decades, routinely depending on combinatorial regimens to achieve effective and durable control of the disease. Proteasome inhibitor, immunomodulatory drug (IMiD), and immunobiologic classes of agents anchor a diverse and expanding therapeutic armamentarium. Commitment to contemporary triplet-based induction regimens and incorporation of risk-adapted maintenance strategies have extended survival, with a median overall survival rate reported at 126.6 months (2). Despite these advances, MM remains an incurable cancer with devastating comorbidity. With no curative treatment options available, development of novel and more effective therapeutics targeting plasma cell biology remains a top priority.

Epigenetic dysregulation represents an important oncogenic mechanism, and histone/protein deacetylases (HDACs) provide a therapeutic target exploitable in MM (3–6). Several nonselective or semiselective HDAC inhibitors, including romidepsin (7, 8), vorinostat (9, 10), and panobinostat (11), have demonstrated varying clinical benefit in combination with bortezomib (BTZ) and dexamethasone for MM. Despite the clinical approval of panobinostat (11), limited efficacy of the pan-HDAC inhibitor has resulted in a failure to carve out a significant niche in MM therapeutic strategies. The development of novel selective HDAC inhibitors would likely improve therapeutic efficacy by interfering more precisely with pathways essential to plasma cell biology and underlying oncologic processes while secondarily minimizing off-target activity contributing to toxicity that can further prevent optimal clinical utility. Recent studies revealed that HDAC11 is overexpressed in MM, correlating with worse prognosis, suggesting this may be an important therapeutic target in this cancer (3).

HDAC11, a relatively uncharacterized HDAC member, is small and structurally distinct; however, it does retain the highly conserved catalytic domain. Recent studies suggest that HDAC11 exhibits efficient fatty acid deacylase activity in comparison to weaker protein deacetylase function lacking histone deacetylase activity required for epigenetic regulation (12–14). Regardless, HDAC11 is now recognized to influence cellular immune function. Sahakian et al. showed that HDAC11 is a critical regulator of neutrophil activation (15). HDAC11 suppresses IL-10 production elicited by LPS stimulation in mouse and human macrophages (16) and was also shown to suppress myeloid-derived suppressor cell expansion and function (17). More recently, HDAC11 was demonstrated to be required for survival and proliferation of JAK2-driven myeloproliferative neoplasms (although dispensable for normal myeloid hematopoiesis) (18). In lymphocytes, HDAC11 influences T cell activation and the transition between naive and memory states (19). Furthermore, HDAC11 deacetylates FOXP3, critical to Treg development and function, importantly demonstrating extended enzymatic function encompassing nonhistone targets (20). However, the role of HDAC11 in the maturation and function of B cells and plasma cells remains unexplored.

Plasma cell differentiation is coordinated by several transcription factors, including IFN response factor 4 (IRF4), B lymphocyte–induced maturation protein 1 (BLIMP1), microphthalmia-associated transcription factor (MITF), paired box 5 (PAX5), X-box binding protein 1 (XBP1), and B cell lymphoma 6 (BCL6) (21). Once activated, B cells ectopically induce IRF4 expression, which is essential for both the initial proliferative burst of activated B cells and induction of plasma cell differentiation (22). Downstream to IRF4, BLIMP1 promotes plasma cell differentiation through repression of B cell–specific genes (23). Together, IRF4 and BLIMP1 interact via a positive feedback loop, which mediates immunoglobulin production and establishment of the full plasma cell transcriptome (24). Additional IRF4 targets, such as SUB1, contribute essential regulatory influences in these transcriptional mechanisms (24). Inappropriately heightened IRF4 activity is a hallmark of myeloma genesis (24). As IRF4 is mutated in less than 5% of patients with MM (25), this hyperactivity relies on an as yet unknown mechanism.

In this study, we demonstrated that HDAC11 expression is critical for plasma cell differentiation and survival. Mechanistically, we have identified a functional interaction between HDAC11 and IRF4 that controls IRF4 acetylation state and influences IRF4-mediated transcriptional functions. Furthermore, human and murine MM cell lines and patient-derived MM cell samples were sensitive to targeted inhibition of HDAC11. Collectively, our findings suggest that HDAC11 interacts with the IRF4/BLIMP1/MYC transcriptional network (24), which is critically dysregulated in myeloma genesis, and HDAC11 inhibition offers a potential therapeutic strategy for MM treatment.

## Results

### HDAC11 regulates plasma cell differentiation.

**Figure 1:**
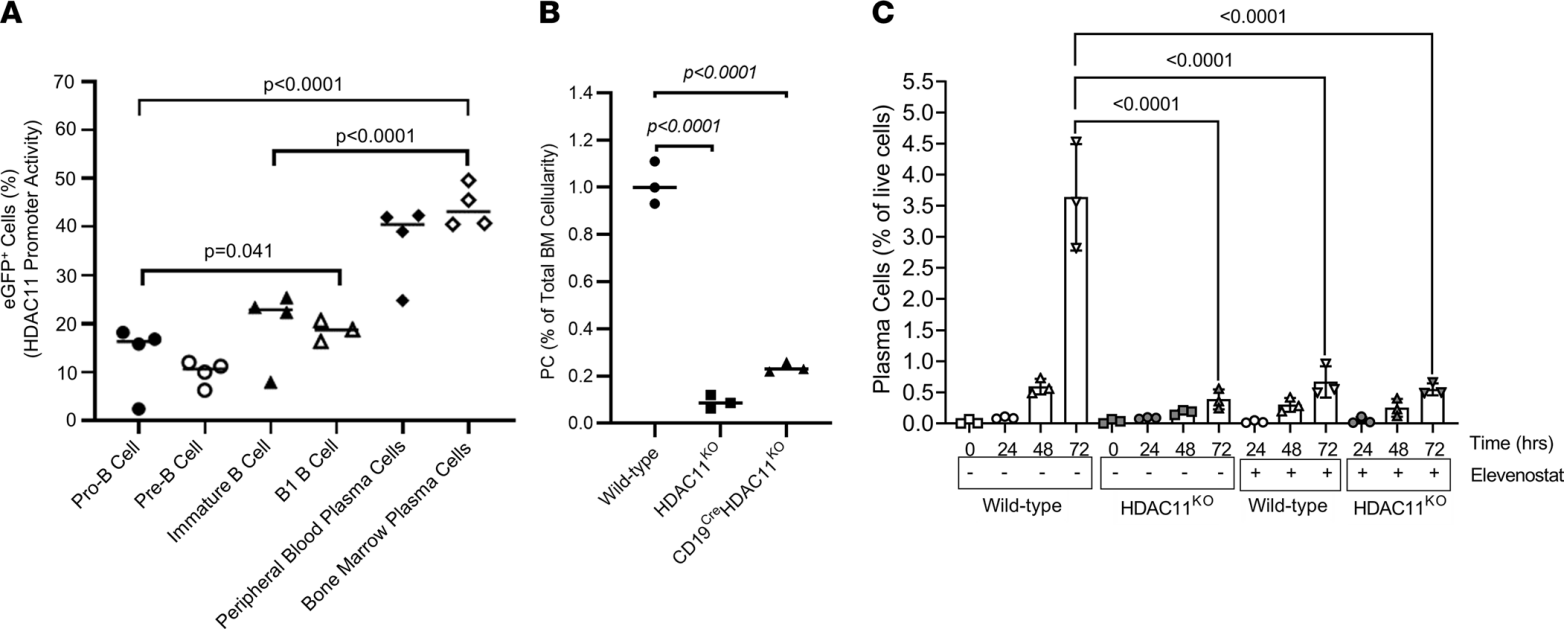
HDAC11 regulates plasma cell differentiation. (**A**) Dynamic visualization of HDAC11 expression in different B cell compartments: pro-B, pre-B, immature/naive, activated B-1, bone marrow plasma cells (BMPCs), and circulating plasma cells (CPCs). The bone marrow and peripheral blood samples were collected from a transgenic reporter mouse (Tg-HDAC11-eGFP) model in which the HDAC11 promoter controls eGFP expression. Bone marrow B cells were isolated from the reporter mice and analyzed by flow cytometry. Stages of B cell development were delineated based on the expression of various cell surface markers, including CD45R (B220), CD19, CD43, and CD138. (**B**) Plasma cell percentages were determined by flow cytometric analysis of bone marrow samples extracted from WT (C57BL/6) and B6.HDAC11^KO^ mice. Loss of plasma cells was demonstrated in 2 HDAC11-deficient mouse strains: the B6.HDAC11^KO^ strain where HDAC11 is globally absent and the C19^cre:LoxP^.HDAC11^KO^ strain with targeted HDAC11 disruption restricted to the B cell lineage. (**C**) Requirement for HDAC11 in plasmablast formation as determined by the in vitro maturation of splenic B cells into plasma cells induced by exposure to mouse IL-4 (1 U/mL), mouse CD40 ligand (0.6 μg/mL), and the TLR agonist Pam3CSK4 (250 ng/mL). Pharmacological inhibition of HDAC11 was achieved by incubating splenic B cells with elevenostat (1 μM). Cells were collected and processed at baseline and at 24, 28, and 72 hours for flow cytometric analyses, and plasma cells were identified as CD19^–^B220^–^CD138^+^ events. Quantitative analysis of plasma cell differentiation entailed calculating average frequency (% of live cells) of viable plasma cells from 3 independent plasmablast assays. Statistical comparisons in all experiments were performed using 1-way ANOVA tests expressed as mean ± SD.

We first observed changes in HDAC11 expression during B cell lymphopoiesis and plasma cell differentiation using a murine transgenic reporter model expressing enhanced GFP (eGFP) driven by the HDAC11 promoter (Tg-HDCA11-eGFP) (26). Although minimally detectable in early B cell ontogeny, eGFP expression was induced in later stages of development with the highest expression detected in plasma cells (Figure 1A), suggesting a significant role for HDAC11 in plasma cell maturation. We confirmed the importance of HDAC11 in plasma cell maturation using 2 different HDAC11-KO mouse models. Mice harboring a global KO of HDAC11 (B6.HDAC11^KO^) exhibited a significant decline (8.7-fold, *P* < 0.0001) in the percentage of bone marrow plasma cells, as shown by flow cytometry (Figure 1B). Similar plasma cell reduction was seen in mice with HDAC11 ablation restricted to B cells via a Cre-LoxP system (CD19^Cre:LoxP^HDAC11^KO^), demonstrating that the effect was intrinsic to the B cell lineage (*P* < 0.0001, Figure 1B).

To look more precisely at the influence of HDAC11 on plasma cell development, we compared plasma cell formation in the presence and absence of HDAC11. Purified splenic B cells from C57BL/6 and B6.HDAC11^KO^ mice were cultured in vitro with TLR agonists in the presence of IL-4 and CD40 antibody ligation to prime plasma cell differentiation. Stimulation of C57BL/6-derived splenocytes induced plasma cell formation in a time-dependent manner, whereas B6.HDAC11^KO^ splenic B cells showed markedly reduced potential to differentiate into plasma cells, and selective inhibition of HDAC11 by elevenostat (ES) produced near-identical results (Figure 1C and Supplemental Figure 1; supplemental material available online with this article; https://doi.org/10.1172/jci.insight.151713DS1). Plasma cell formation was not further suppressed when ES was added to B6.HDAC11^KO^-derived B cells, affirming ES specificity for HDAC11. Overall, these observations indicate that efficient plasma cell formation requires HDAC11.

### HDAC11 interacts with IRF4.

**Figure 2:**
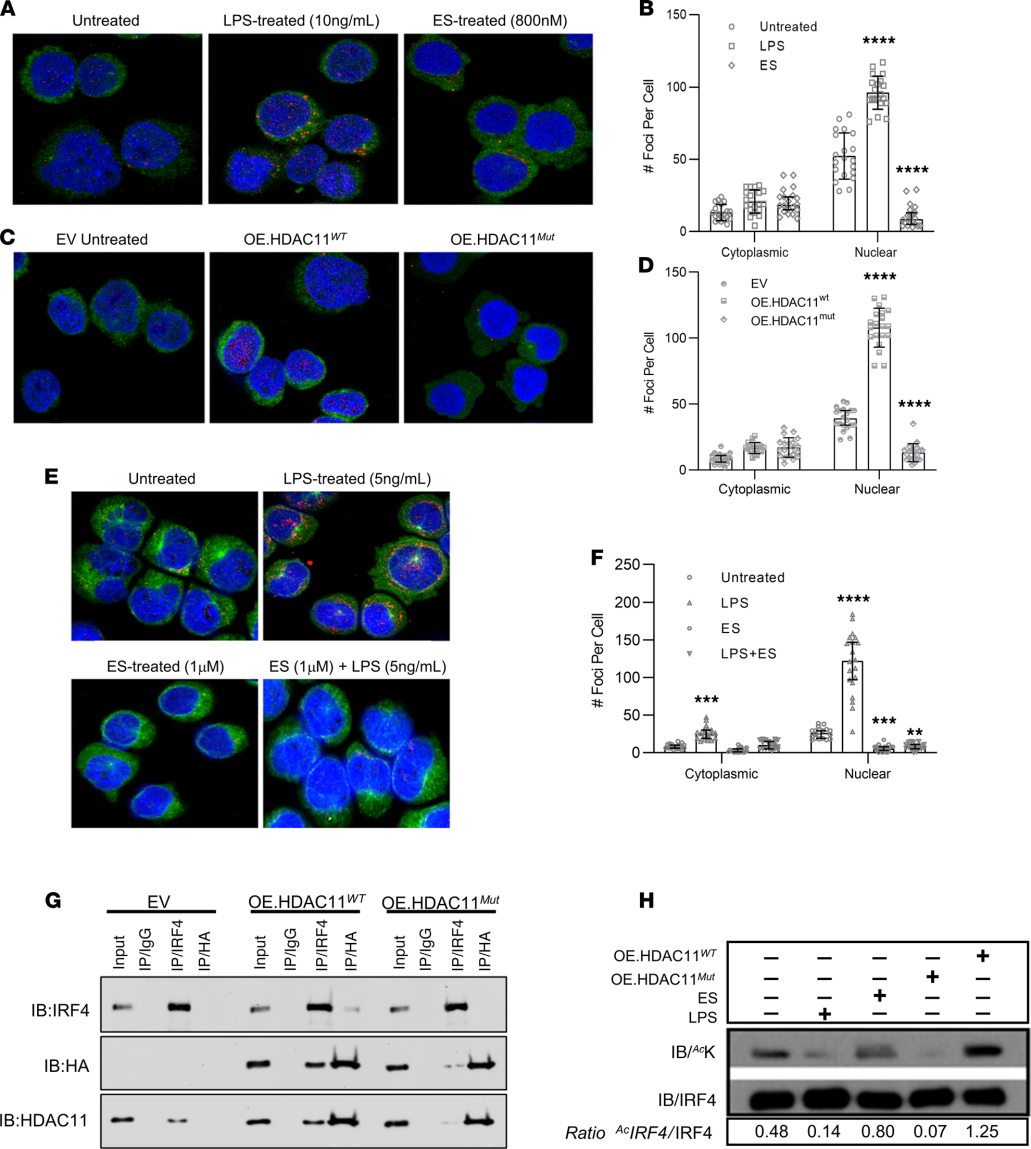
HDAC11 interacts with IRF4 and regulates IRF4 acetylation status. (**A**–**D**) PLA was performed on MM1.S cells. (**A**) Parental MM1.S cells were conditioned with LPS (5 μg/mL) or elevenostat (ES; IC_50_ dose). (**C**) MM1.S cells were transfected with plasmid constructs containing WT or enzymatically inactive HDAC11 or an empty vector as a control. PLA signals (red fluorescence) were detected by confocal microscopy. DAPI provided nuclear counterstaining and Alexa Fluor 488–labeled α-tubulin provided cytoplasmic counterstain. (**B** and **D**) Quantitative analysis of PLA signals, analyzed by 2-way ANOVA reported as mean ± SD. (**E**) PLA on primary MM cells derived from patient samples. Cells were cultured with the serum collected from the same patient and incubated with LPS (5 μg/mL) and ES (1 μM) or combination of LPS and ES (24 hours), ×40 magnification. (**F**) Quantitative analysis of PLA signals in the primary MM cells presented as median with 95% CI. Statistical comparison was performed using a 2-way ANOVA test with data expressed as mean ± SD; **P < 0.005; ***P < 0.0005; ****P < 0.0001. (**G**) Reciprocal co-IP assays conducted on MM1.S cell lines stably transfected with an HA-tagged WT or enzyme-inactive version of HDAC11, empty vector–transfected cells were used as control. IP performed with anti-IRF4 and anti-HA antibody, rabbit IgG (rIgG) used as isotype control; gel image representative of experiment run in triplicate. (**H**) Western blot detection of acetylated lysine (^Ac^K) after IP of IRF4. Cell lysates were immunoprecipitated with an anti-IRF4 antibody followed by immunoblotting with antibodies against ^Ac^K or IRF4; IRF4 acetylation was quantified based on the ratio of acetylated IRF4 to total IRF4 (^Ac^IRF4/IRF4); image presents results of 1 of 3 independent experiments.

The developmental blockade imposed by the absence or inhibition of HDAC11 pointed to a mechanism involving interference with the genetic programing guiding plasma cell maturation. Given that IRF4 is a quintessential transcriptional regulator of plasma cell biology, we were curious whether HDAC11 might be important for optimal IRF4 activation. Notably, HDAC11 has been shown to deacetylate the transcription factor FoxP3 in T cells (20), raising suspicion that IRF4 could be a direct enzymatic substrate of HDAC11. We therefore tested the hypothesis that HDAC11 interacts with IRF4. Proximity ligation assay (PLA) provided quantifiable verification of the HDAC11/IRF4 interaction in MM1.S cells. As shown in Figure 2 A–D, HDAC11/IRF4 complexes were visible predominantly in the nuclear compartment of MM1.S cells. We detected an increase in HDAC11/IRF4 interactions in MM1.S cells upon LPS exposure, which induces activation and proliferation in MM cells (27–29). Overexpression of functionally intact HDAC11 (HDAC11^wt^) similarly increased HDAC11/IRF4 interactions in MM1.S cells. In contrast, treatment with ES disrupted HDAC11/IRF4 interactions, as did overexpression of a mutated HDAC11 (HDAC11^mut^) construct incorporating alterations in the enzymatic binding domain to disrupt deacetylase activity. HDAC11/IRF4 complexes were also visualized in patient-derived primary MM cells (Figure 2, E and F). As in the MM1.S cell line, LPS treatment of primary MM cells increased nuclear complex formation 4.5-fold, whereas ES exposure resulted in a depletion of nuclear HDAC11/IRF4 interactions. LPS was unable to overcome the effects of ES in primary MM cells upon simultaneous exposure (Figure 2, E and F). HDAC11/IRF4 interactions could also be readily detected upon co-IP as well, with reciprocal detection after IP with either IRF4 or HDAC11 (Figure 2G). Importantly, the interaction was attenuated in cell lines expressing the enzymatically inactive HDAC11 (Figure 2G). This HDAC11/IRF4 interaction implies a direct regulatory function.

### HDAC11 deacetylates IRF4.

Transcription factor acetylation states influence transcriptional activity (20, 30). Although acetylation was not previously known to regulate IRF4, the demonstration of HDAC11/IRF4 interactions led us to hypothesize that acetylation is an important regulatory mechanism for the IRF4 signal pathway. Interestingly, IRF4 mutations replacing lysine (K) residues, which serve as potential acetylation sites, have been reported in approximately 3% to 4% of patients with newly diagnosed MM (25, 31, 32). Consistent with this, 25 of 513 (4.9%) patients screened at our institution (33) harbored an IRF4 mutation, with the majority being a specific K123R mutation and much less frequently, a K59R mutation. However, mass spectrometry analyses of the IRF4 peptide sequence encompassing 90% coverage failed to detect acetylation events at lysine residues at position 123 (K^123^) or position 59 (K^59^) (Table 1), suggesting that the most frequently observed mutations in IRF4 seen in patients with MM did not directly alter acetylation targets. Importantly, mass spectrometry mapping revealed a single IRF4 acetylation site uniquely at a lysine corresponding to position 103 (K^103^) adjacent to the nuclear localization sequence. IRF4 acetylation at K^103^ was confirmed with targeted mass spectrometry methods (Table 1 and Supplemental Figure 2). Changes in IRF4 acetylation were confirmed by IP of IRF4 followed by immunoblotting with an antibody recognizing acetylated lysine (Figure 2H). Furthermore, induction of HDAC11 by LPS stimulation or overexpression of HDAC11^wt^ in MM1.S cells decreased acetylation of IRF4. In contrast, inhibition of HDAC11 or overexpression of an enzyme-inactive HDAC11^mut^ resulted in IRF4 hyperacetylation. Collectively, these results indicate that HDAC11 promotes the deacetylation of IRF4, unveiling a potentially novel regulatory mechanism controlling IRF4 activity.

### HDAC11 inhibition suppresses IRF4 transcriptional function.

**Figure 3:**
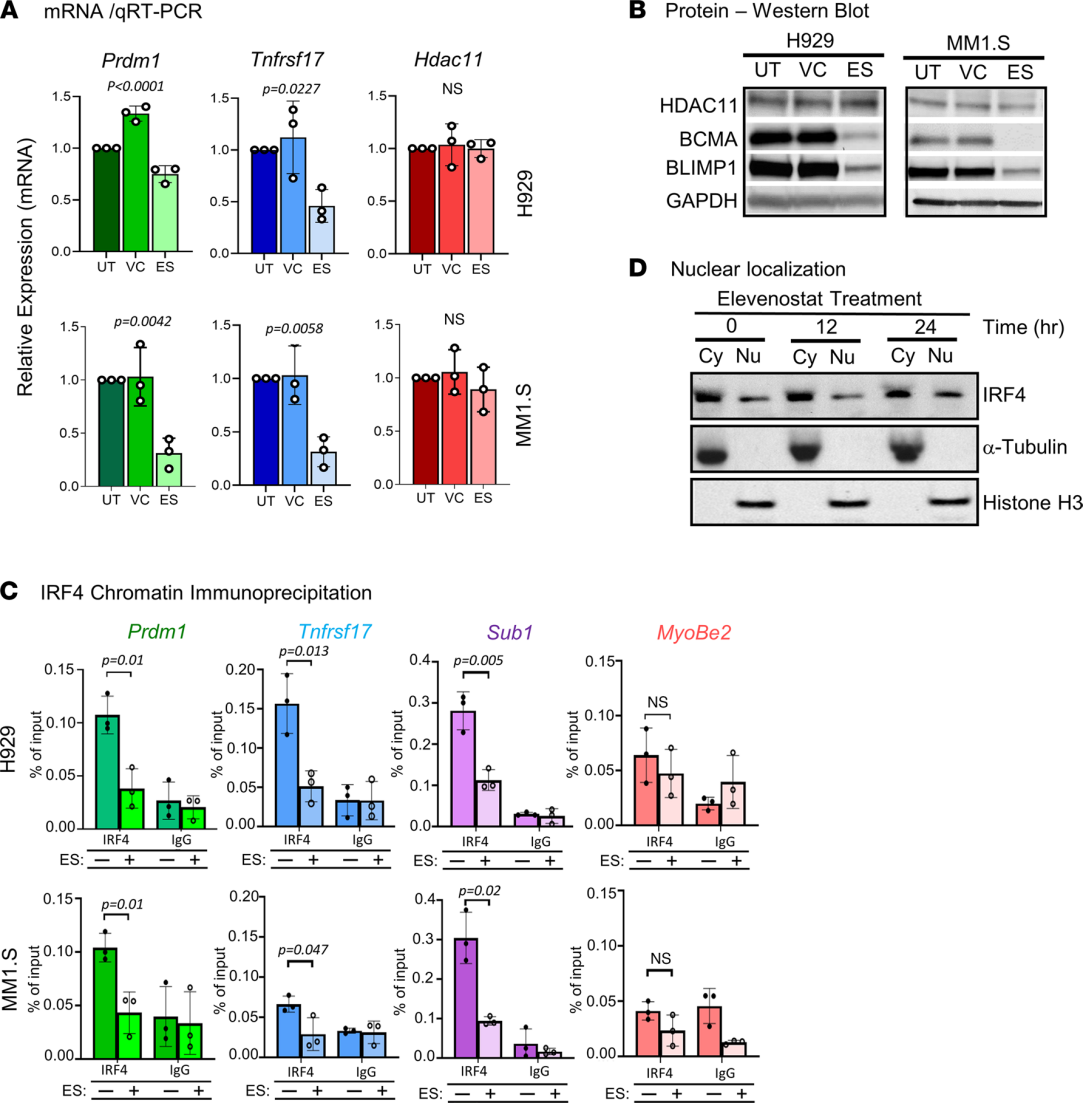
HDAC11 regulates IRF4 transcriptional function. (**A**) Relative mRNA levels of *PRDM1*/Blimp-1, *TNFRSF17*/BCMA, and *HDAC11* in MM1.S and H929 cells treated with ES; expression determined by real-time qPCR, normalized to housekeeping gene GAPDH. The data, representing 3 independent experiments, are presented relative to the untreated (UT) and DMSO vehicle control (VC), ES: treated with elevenostat for 24 hours. Data are expressed as mean ± SD, calculated using a 1-way ANOVA test. (**B**) Protein levels of BLIMP-1, BCMA, and HDAC11 in MM1.S and H929 cells treated with ES for 24 hours; gel image represents results of 1 of 3 independent experiments. (**C**) IRF4 binding to the *PRDM1*, *TNFRSF17*, *SUB1,* and *MYOBE2* (negative control) promoters was determined using ChIP–qPCR. ChIP was performed using an anti-IRF4 antibody on chromatin derived from MM1.S and H929 cell lines treated with ES or DMSO. Values are presented as percentage of input, calculated as normalized signal from immunoprecipitated material divided by input DNA signal (pre-IP) in arbitrary units; data represents 3 independent experiments performed in each cell line. (**D**) HDAC11 inhibition resulted in no changes in subcellular localization of IRF4. MM1.S cells were treated with ES (750 nM) for 24 hours and cell lysates were then prepared with cytosolic and nuclear fractionation; figure is representative of experiment performed in triplicate; data reported as mean ± SD with statistical analyses performed using 1-tailed Student’s *t* test. Cy, cytosolic fraction; Nu, nuclear fraction.

The acetylation site in IRF4 resides proximal to several important functional domains, including the putative DNA binding domains and nuclear localization sequence, suggesting potential mechanistic roles for IRF4 acetylation. Lending support to this hypothesis, treatment of MM1.S and H929 cells with ES (0.5 × IC_50_ dose) resulted in marked downregulation of *PRDM1*/BLIMP1 and *TNFRSF17*/B cell maturation antigen (BCMA), known targets of IRF4 transcriptional regulation. *PRDM1* (MM1.S, *P* = 0.011; H929, *P* = 0.006) and *TNFRSF17* (MM1.S, *P* = 0.0001; H929, *P* = 0.004) mRNA levels decreased within 24 hours, determined using real-time quantitative reverse transcription (qRT-PCR) (Figure 3A), with similar decreases in corresponding protein levels (Figure 3B). Depletion of HDAC11 in MM1.S cells using a transient siRNA interference technique showed similar changes in IRF4 target genes (Supplemental Figure 3). Examination of IRF4 occupation of the promoter regions of *PRMD1* and *TNFRS17* as well as *SUB1* demonstrated marked reduction in IRF4 binding after incubation with ES (*PRDM1*: MM1.S, *P* = 0.01; H929, *P* = 0.01; *TNFRSF17*: MM1.S, *P* = 0.001; H929, *P* = 0.013; *SUB1*: *P* = 0.02; H929, *P* = 0.005), whereas no change was seen in the control *MYOBE2* target (MM1S, NS; H929, NS) (Figure 3C). Importantly, *PRDM1* and *TNFRSF17* are important factors in plasma cell differentiation and survival, respectively. The rapid loss of expression of these genes after inhibition of HDAC11 activity provides further insight into the impaired plasma cell maturation observed in Figure 1C. Despite the loss of IRF4 occupation at well-established target gene promoter regions and subsequent loss of target gene expression, there did not appear to be any depletion of IRF4 in the nucleus within the same 24-hour time frame (Figure 3D). Collectively, the resulting decrease in IRF4 transcriptional activity upon HDAC11 inhibition implies that HDAC11 shares a functional relationship with IRF4. And given that interference with IRF4 was previously shown to impair plasma cell survival (24, 34), these results suggest that HDAC11 inhibition produces similar effects.

### HDAC11 expression in myeloma.

**Figure 4:**
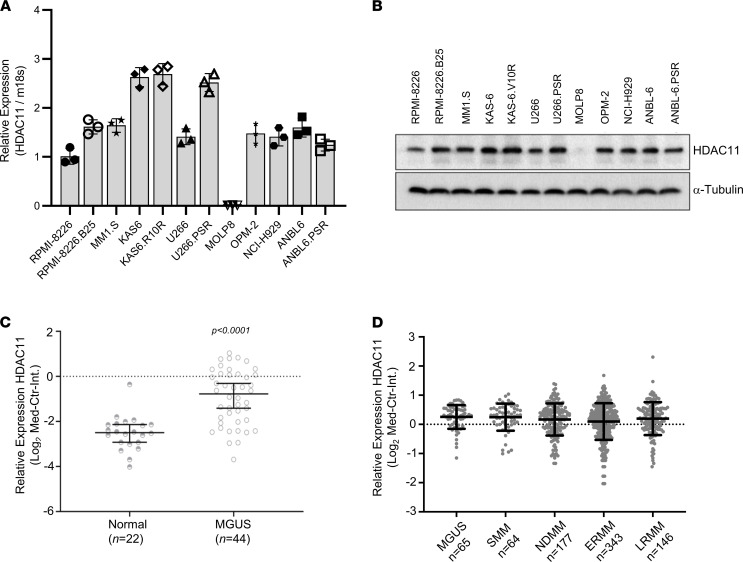
HDAC11 expression in myeloma. (**A**) Quantitative expression of HDAC11 in 12 MM cell lines by real-time qPCR. HDAC11 expression levels were normalized to 18 subunit rRNA expression in corresponding cell lines; figure is representative of 3 independent experiments. (**B**) HDAC11 protein levels in 12 MM cell lines determined by Western blot. (**C**) Analysis of the Zhan et al. data set available by public accessed via Oncomine (http://www.ncbi.nlm.nih.gov/geo/query/acc.cgi?acc=GSE5900) showed significant upregulation of HDAC11 in MM precursor state, MGUS (monoclonal gammopathy of undetermined significance) relative to healthy donors; statistical analysis performed using 1-tailed Welch’s *t* test and reported as mean ± SD. (**D**) HDAC11 expression was compared in bone marrow–derived CD138 selected cells from 65 MGUS, 64 smoldering or asymptomatic multiple myeloma (SMM), 177 symptomatic newly diagnosed multiple myeloma (NDMM), 343 early relapsed refractory multiple myeloma (ERMM), and 146 late relapsed refractory multiple myeloma (RRMM); presented as log_2_ median-centered intensity (Med-Ctr Int) with 95% CI.

Because therapeutic efficacy of any agent is dependent on consistent expression of the drug target, we first sought to assess HDAC11 expression levels in MM. HDAC11 expression was detected at relatively consistent levels in cell lysates prepared from 11 of 12 human MM cell lines as demonstrated by qRT-PCR detection of mRNA (Figure 4A) and Western blot detection of protein (Figure 4B). The MOLP8 cell line was the exception, with little to no detectable HDAC11. Interrogation of a publicly available RNA expression data set (35) demonstrated that *HDAC11* expression was significantly higher in plasma cells from individuals with monoclonal gammopathy of undetermined significance (MGUS) relative to healthy donors (Figure 4C; MGUS [*n* = 44] vs. healthy donors [*n* = 22], *P* < 0.0001). We further identified a marked consistency of *HDAC11* mRNA expression across MM disease states relative to MGUS in the interrogation of RNA-Seq from 65 patients with MGUS, 64 patients with smoldering MM, 177 patients with newly diagnosed MM, 343 patients with early relapsed/refractory MM (RRMM, 1–3 prior lines of therapy), and 146 patients with late RRMM (>3 lines of therapy) (Figure 4D). Collectively, HDAC11 expression was increased in plasma cell dyscrasias as compared with plasma cells isolated from healthy donor marrow samples, but expression was largely consistent throughout plasma cell disorders even in the face of therapy resistance. This would suggest that HDAC11 could represent a targetable vulnerability in all MM states.

### HDAC11 inhibition demonstrates anti-myeloma activity.

**Figure 5:**
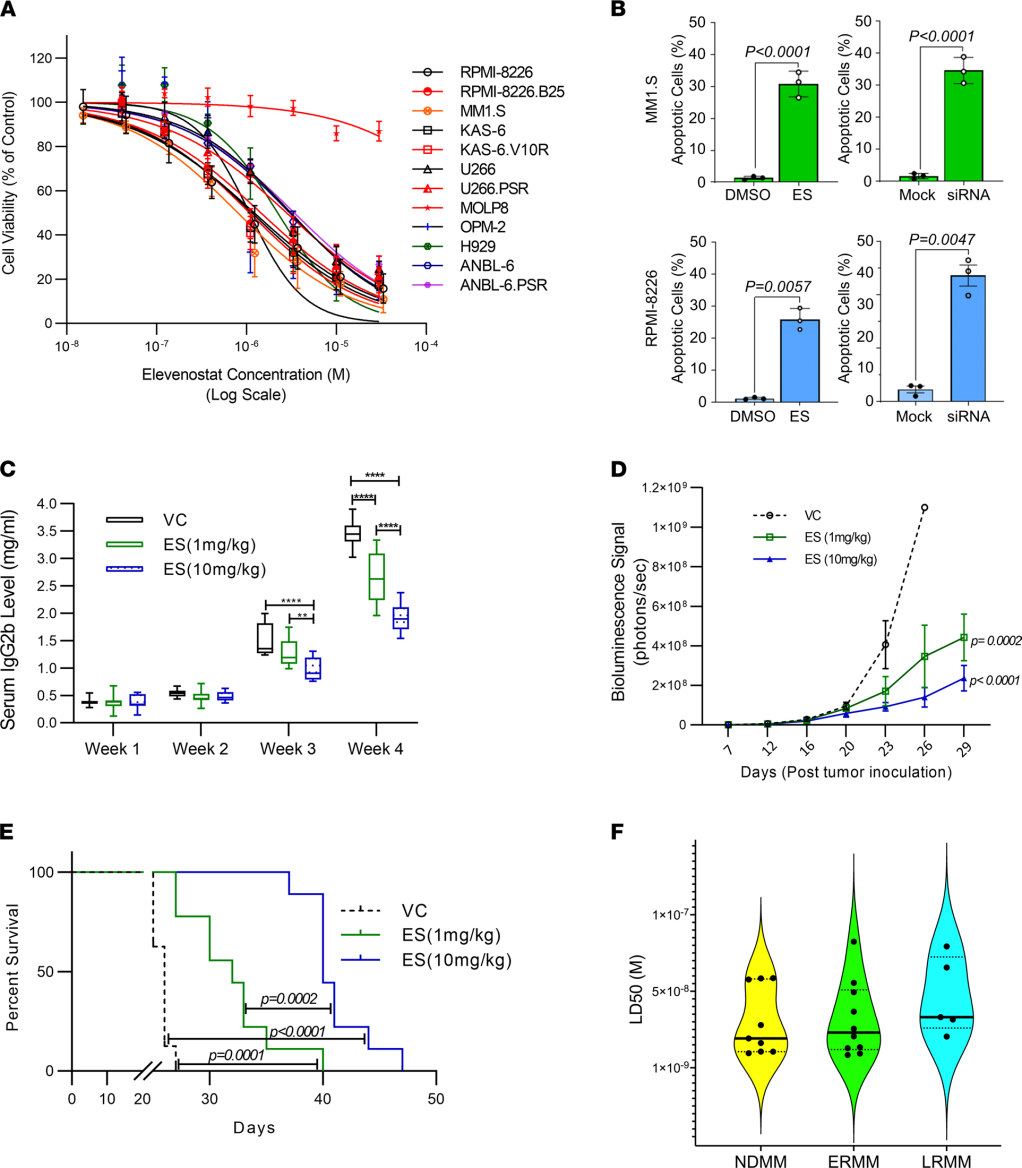
HDAC11 inhibition demonstrates anti-myeloma properties. (**A**) Cytotoxic effect of elevenostat (ES) in 12 different myeloma cell lines. Cells were treated with ES for 72 hours at the indicated concentrations, and viability was determined with CCK-8 assay; representative of 3 independent experiments with each cell line. (**B**) Caspase-3 activation in MM1.S and RPMI-8226 cells treated with ES (750 nM) versus DMSO or transiently transfected with HDAC11 or nontargeting siRNA. Caspase-3 activation was assessed by flow cytometry at 48 hours (ES) or 72 hours (siRNA). Statistical significance was determined by 1-tailed Welch’s *t* test calculated on 3 independent experiments. (**C**) Serum IgG2b levels (measured on a weekly basis) in the blood samples of C57BL/KaLwRij mice challenged with 5TGM1-luc MM cells. Results are presented as mean ± SD and statistical analysis computed using a 2-way ANOVA test comparing vehicle control (VC), ES 1 mg/kg/day (ES 1 MKD), and ES 10 mg/kg/day (ES 10 MKD); *n* = 11 per group. **P < 0.005; **P < 0.0001 (**D**) Quantitative analysis of the bioluminescence imaging (performed twice per week), presented as mean ± SD computed using a 2-way ANOVA test. (**E**) Survival of mice is shown as Kaplan-Meier curves. The experiment was terminated after 47 days. Log-rank test was used for statistical comparison. Each group contained 9 mice (*n* = 9). (**F**) LD_50_ values of ES in primary myeloma patient samples obtained from newly diagnosed (ND), early relapsed refractory (ERR), and late relapsed refractory (LRR) patients.

Since MM cell survival requires IRF4 (24), we next examined whether HDAC11 inhibition also diminishes MM cell survival. ES exposure showed profound cytotoxicity in human MM cell lines (Figure 5A; IC_50_ values range 0.803–3.410 μM). Importantly, MOLP8 cells, which expressed minimal HDAC11 compared with other MM cells lines in our analysis (Figure 4, A and B), were resistant to ES-mediated cytotoxicity. As a typical example, MM1.S cells underwent apoptotic cell death, with 31.4% and 34.3% of cells expressing activated caspase-3, detected by flow cytometry, after exposure to ES for 48 hours or 72 hours after transfection with siRNA depleting *HDAC11*, respectively (Figure 5B and Supplemental Figure 4). Treatment of RPMI-8226 cells similarly with ES or *HDAC11* siRNA produced comparable results, inducing caspase-3 activation in 29.4% and 53.0%, respectively (Figure 5B and Supplemental Figure 4). ES also elicited potent dose-dependent in vivo tumor suppression in the immune-competent 5TGM1/C57BL/KaLwRij mouse MM model, as revealed by the suppression of serum IgG_2b_ levels (Figure 5C). By week 3, average serum IgG_2b_ levels decreased 23.23% (*P* < 0.0001) and 44.86% (*P* < 0.0001) in response to ES 1 mg/kg/day and 10 mg/kg/day, respectively. Tumor control was confirmed by bioluminescence imaging (Figure 5D). Accordingly, ES treatment led to a significant survival benefit in 5TGM1-*luc*–bearing mice (Figure 5E). The median survival for vehicle-treated mice was 26 days. In contrast, survival for ES-treated mice extended to 32 days in the low-dose 1 mg/kg/day group and to 40 days in the high-dose 10 mg/kg/day group. ES was well-tolerated at these doses and dosing frequencies, supported by lack of changes in body weight (Supplemental Figure 5). Lastly, we examined the activity of ES in CD138-selected MM cells isolated from 34 patient bone marrow samples (newly diagnosed MM, early RRMM, and late RRMM) using an ex vivo organoid model system designed to assess drug sensitivity in patient-derived MM samples that incorporates essential elements of the bone marrow tumor microenvironment as previously described (36–38). ES was equally effective in newly diagnosed MM and RRMM patient samples, with an LD_50_ value of 38.22 nM (Figure 5F, range 9.3–83.4 nM), suggesting that HDAC11 remains a vulnerability in both early- and late-stage MM.

### HDAC11 synergizes with proteasome inhibitors in vitro and ex vivo.

**Figure 6:**
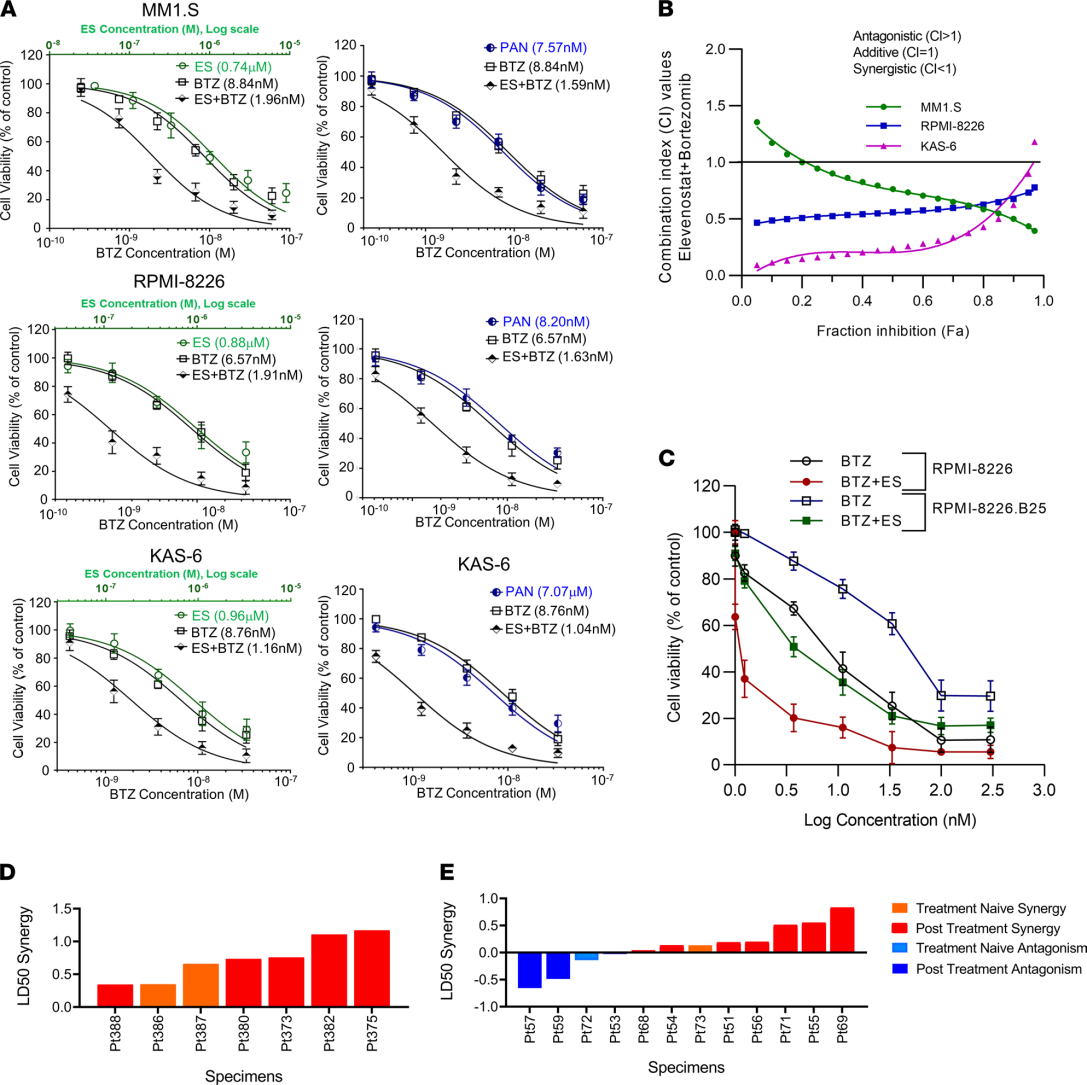
HDAC11 synergizes with proteasome inhibitors in vitro and ex vivo. (**A**) HDAC11 inhibition via ES potentiated the cytotoxic effects of BTZ in myeloma cell lines. Cells were cultured in presence of dose ranges of ES and BTZ. For combination treatment, ES and BTZ were combined at a ratio of 100:1; BTZ and PAN were combined at a 1:1 ratio. Cytotoxic effect at 72 hours was measured by CCK-8 assay. The IC_50_ values were calculated using nonlinear regression model and presented in the graph. All graphs are representative of 3 replicate assays. (**B**) The synergistic potential of combining ES with BTZ was assessed in 3 cell lines (MM1.S, RPMI-8226, and KAS-6) by following the Chao-Talalay method (31). Combination index (CI) values of drug combination were determined by CompuSyn software based on the cytotoxicity data obtained from CCK-8 assay (graph representative of 3 separate experiments). (**C**) The IC_50_ values of single-agent and combination drug treatments on parental and resistant cell lines were determined by using nonlinear regression (curve fit) analysis (GraphPad Prism) based on the cytotoxicity data obtained from CCK-8 assay; data representative of 3 independent experiments. The synergy between ES and BTZ (**D**) and PAN and BTZ (**E**) in comparison was evaluated by comparing experimentally derived combined-drug sensitivity to mathematical additivity derived from single-agent sensitivity, measured according to LD_50_. Each bar represents the degree of synergy (orange) or antagonism (blue) assessed in an individual patient sample. ES, elevenostat; BTZ, bortezomib; PAN, panobinostat.

Recognizing the excellent single-agent activity of ES in MM cell lines, we next examined combination activity between BTZ and ES in vitro and ex vivo. ES enhanced BTZ cytotoxic potential 4.5-fold in MM1.S, 3.5-fold in RPMI-8226, and 7.5-fold in KAS-6 cells (Figure 6A). Synergy between ES and BTZ was verified using the Chao-Talalay method and CompuSyn software, indicating synergy by a combination index less than 1 (Figure 6B). In addition, ES was able to restore BTZ sensitivity in the BTZ-resistant RPMI-8226.B25 cells (Figure 6C), and this resensitization was replicated in KAS-6.V10R cells also resistant to BTZ (data not shown). This synergy between ES and BTZ was similarly observed in patient-derived MM cells ex vivo cultured in the presence of patient-derived stromal elements to recapitulate essential elements of the bone marrow microenvironment; in comparison, panobinostat-BTZ synergy was detected less consistently (Figure 6, D and E; and Supplemental Figure 6) (39).

## Discussion

Our data demonstrated a potentially novel role for HDAC11 as a critical regulator of plasma cell differentiation and survival. Interference in HDAC11 function dramatically decreased B cell maturation into plasma cell cells in vitro upon activation. Mechanistically, HDAC11 promotes plasma cell development and survival by regulating the activity of IRF4. Interference with HDAC11 activity results in hyperacetylation of IRF4 and a concomitant decrease in IRF4 transcriptional function demonstrated by the loss of IRF4 at known target promoter sites and diminished expression of *PRDM1* and *TNFRSF17* genes known to be positively regulated by IRF4. HDAC11 inhibition, likely via its inactivation of IRF4, delivers a cytotoxic insult to myeloma cells in vitro. This translates to improved survival in a well-defined in vivo murine myeloma model, and anti-MM potential was also consistently reproduced in primary MM samples isolated from fresh patient bone marrow biopsies, an activity that was synergistic with BTZ. The observed decreases in *PRDM1*/BLIMP1 and *TNFRSF17*/BCMA resulting from HDAC11 inhibition of IRF4 provide a reasonable explanation for the impaired plasma cell development and cytotoxic effects observed on MM cells. BLIMP1 is important for plasma cell maturation and longevity (40–42), and BCMA provides an important survival signal for plasma cells (43).

Multiple HDAC inhibitors have been evaluated for efficacy in MM, but their performance in the clinical setting has thus far failed to meet expectations (7, 9–11, 44). Panobinostat, the first-in-class HDAC inhibitor approved for use in treatment of MM, is a potent nonselective inhibitor. Although lacking meaningful single-agent clinical activity, panobinostat was demonstrated to overcome resistance to proteasome inhibitors (45). However, excessive toxicity, including black box warnings related to gastroenteric and cardiac side effects, likely caused by potent inhibition of HDAC isoforms inconsequential in plasma cell and MM biology, has restricted effective utilization clinically. Second-generation HDAC inhibitors have been designed to minimize this off-target toxicity by selectively targeting isoforms contributing to the disease biology. Ricolinostat, a selective HDAC6 inhibitor, emerged as the first selective HDAC inhibitor based on its ability to interfere with the aggresome pathway believed to contribute to proteasome inhibitor resistance (44, 46). Ricolinostat was ineffective in combination with BTZ (44) but arguably more effective in combination with lenalidomide (47), suggesting that targeting mechanisms of drug resistance may represent a flawed strategy. In contrast, we have demonstrated that the selective inhibition of HDAC11 promoted the deacetylation of IRF4 — a vital signal pathway essential to the biology and oncogenic mechanism of plasma cell myeloma — and may provide a more effective translational strategy.

The exact mechanism of regulation will require additional verification, but HDAC11 appears to control the acetylation of IRF4, altering the capacity of IRF4 to promote plasma cell differentiation and viability. However, we have yet to determine whether this is via a direct or indirect mechanism. Studies in T cells unveiled that deacetylation of the transcription factor FOXP3 was similarly dependent on the formation of a transcription factor/HDAC11 complex, although a direct enzyme/substrate relationship was not conclusively established (20). HDAC11 has been shown to act as a more potent fatty-acid deacylase relative to its histone deacetylase activity (12, 13), raising the possibility that HDAC11 may regulate IRF4 acetylation via the indirect recruitment of an additional protein deacetylase to the transcriptional complex. However, in support of a potential direct regulatory role, we showed that the mutant variant of HDAC11, which selectively interrupts the enzymatic pocket structure (48), resulted in IRF4 hyperacetylation and prevented the interaction between HDAC11 and IRF4 as seen by PLA and co-IP. These results suggest that IRF4 is, in fact, deacetylated by HDAC11.

IRF4 mutations are found in approximately 3% to 4% of patients with newly diagnosed MM (25, 31, 32), and K123R missense mutations comprise the majority of these events. Despite the close proximity of K^123^ to the functional domain determining PU.1 transcription cofactor interaction (49), K^123^ does not appear to be an acetylation target responsible for the acetylation-dependent regulation of IRF4 that we observed. A second previously reported lysine-targeting missense mutation, K59R, which resides proximally to the DNA binding domain (49), similarly does not appear to be an acetylation target. Review of genomic data from 513 patients treated at our institution detected IRF4 mutations in 4.9% of screened samples, but none of these involved the lysine at position 103. This was at first surprising because ablation of the acetylation site would be predicted to enhance IRF4 activity as is seen in MM. However, IRF4 hyperactivity seen in MM plasma cells is driven by the aberrant activation of a positive feedback loop involving IRF4 and c-MYC, resulting in the enforced overexpression of both factors (24). The presence of a more potent induction of IRF4 via genetic regulation may neutralize any selectable advantage introduced by a point mutation at K^103^, resulting in the eradication of the acetylation site serving as a suppressive regulatory switch. Importantly, the lack of mutations at this K^103^ site implies that acetylation-mediated regulatory mechanisms for IRF4 remain intact, and promoting the hyperacetylation of IRF4 may represent an effective means of dampening the oncological effects of the aberrantly overactive MYC-IRF4 circuit.

Aberrant IRF4 and c-MYC activation are a hallmark of the oncogenic process underlying MM pathogenesis. The success associated with IMiDs in the treatment of MM is in part defined by the ability of agents in this drug class to elicit the downmodulation of IRF4 and c-MYC (50–52). IMiDs, including lenalidomide, pomalidomide, and thalidomide, interact with cereblon (CRBN), the substrate recognition component of the CRBN-CRL4 E3 ubiquitin ligase complex, thereby directing the degradation of IKAROS and AIOLOS (53–55) and subsequent decreases in IRF4 and c-MYC (52). Given the activity of IMiDs in MM, there is reason to assume that targeted inhibition of HDAC11, acting similarly to suppress IRF4, could possess similar therapeutic potential. Furthermore, inhibition of IRF4 by dual independent mechanisms offers a potential synergistic effect, or alternatively a therapeutic option to overcome IMiD resistance. Interestingly, the genetic ablation of HDAC11 is associated with a heightened level of T cell activation (19), offering the possibility that, similar to agents of the IMiD class, HDAC11 inhibition may also enhance anti-MM T cell immune responses.

Interference with HDAC11 has previously been demonstrated to affect immune cell function in both lymphoid and myeloid compartments. As previously noted, T cells in HDAC11-deficient mice exhibit enhanced proliferation, stimulated cytokine production, and engagement of cytotoxic effector mechanisms (19). HDAC11^KO^ T cells introduced into recipient mice by adoptive transfer displayed heightened reactivity, translating to resistance to tolerance, enhanced antitumor activity, and more severe graft-versus-host disease (GvHD) in an allotransplant model. The targeted interruption of HDAC11 in FoxP3^+^ Tregs resulted in more potent suppressive function and in vivo administration of ES, where in this case HDAC11 interference was not isolated to a specific cell population, prevented allograft rejection in MHC-mismatched mice (20). HDAC11 also appears to exert a regulatory role in cells of the myeloid lineage. In part, this reflects the ability of HDAC11 to suppress IL-10 expression (16). Disruption of HDAC11 in antigen-presenting cells resulted in diminished T cell activation (16), while myeloid-derived suppressor cells lacking HDAC11 more potently inhibited antigen-specific T cell activation (17). However, loss of HDAC11 in neutrophils conversely correlates with an increase in migratory and phagocytic capacity (15). With the success of therapeutics targeting both the tumor and cellular immune effectors, it will be important to define the immunological impact of HDAC11 inhibitors because this may complement the observed anti-MM activity.

In summary, we have demonstrated that HDAC11 is central to orchestrating the transformation of an activated B cell into a plasma cell and that HDAC11 regulates the transcriptional activity of multiple genes essential to plasma cell and myeloma cell proliferation and survival. Moreover, HDAC11 accomplishes this through direct interaction with the IRF4 transcription factor, which has a well-established role in controlling B cell and plasma cell differentiation as well as myeloma genesis. Interference with HDAC11 function results in a hyperacetylated state of IRF4. Observed decreases in the occupation of the *PRDM1*, *TNFRSF17,* and *SUB1* promoter regions, known IRF4 target genes, may be the result of the destabilization of IRF4 interactions required for transcriptional complex formation or for DNA binding. Collectively, these observations point to an intriguing translational potential and strongly attest to the need for further investigation of selective HDAC11 inhibition as a potential therapeutic option for MM.

## Methods

### Mice.

C57BL/6 WT mice were purchased from Charles River Laboratories. Tg-HDAC11-eGFP transgenic reporter mice expressing eGFP driven by the HDAC11 promoter (26, 56) were obtained from Nathaniel Heintz through the Mutant Mouse Regional Resource Centers. B6.HDAC11^KO^ mice lacking HDAC11 expression either entirely or CD19^cre:LoxP^.HDAC11^KO^ lacking HDAC11 selectively in the B cell lineage (15) were obtained from Eduardo Sotomayor’s and Ed Seto’s labs, respectively (George Washington University, Washington, DC, USA).

### Cell lines and patient samples.

Except as noted, all human MM cell lines were originally purchased from American Type Culture Collection. Murine MM cell lines 5TGM1 and 5TGM1.*luc* (luciferase-transfected 5TGM1) were obtained from University of Texas Health Science Center (San Antonio, TX). KAS-6.V10R and ANBL-6.V10R, BTZ-resistant subclones of the KAS-6 and ANBL-6 parental cell lines (57), were provided by Robert Z. Orlowski (MD Anderson Cancer Center, Houston, TX); U266.PSR, a BTZ-resistant variant of the U266 parental cell line (58), was obtained from Steve Grant (Virginia Commonwealth University, Richmond, VA). Bone marrow samples were obtained from patients with MM treated at Moffitt Cancer Center and collected according to the Total Cancer Care clinical study protocol.

### Antibodies and reagents.

ES, a selective HDAC11 inhibitor (20), was purchased from BioVision. LIVE/DEAD Fixable NIR was purchased from Thermo Fisher Scientific, and the fluorescent-conjugated antibodies allophycocyanin (APC) α-CD19 (clone 6D5), BV421α-CD138/Syndecan-1 (clone 281-2), and Alexa Fluor 488 (AF488) α-CD45R/B220 (clone RA3-6B2) monoclonal antibodies were purchased from BioLegend. Phycoerythrin (PE) α-active caspase-3 antibody (clone C92-605) was purchased from BD Biosciences Pharmingen. Unless otherwise mentioned, all other antibodies for flow cytometry were purchased from BD Biosciences. Western blot experiments used antibodies detecting BCMA, BLIMP1, IRF4, α-tubulin, histone H3, and HA (all purchased from Cell Signaling Technology) and HDAC11 (Novus Biologicals).

### Plasma cell and B cell lineage maturation analyses.

Flow cytometry analyses of PBMCs, bone marrow aspirates, and splenocytes were performed using fluorochrome-labeled monoclonal antibodies V450α-CD3 (clone 500A2), V450α-NK1.1 (clone PK136), PE-Cy7α-CD45 (clone 30-F11), APC-Cy7α-CD19 (clone 1D3), APCα-CD43 (clone 1G10), and PEα-CD138 (clone 281-2), all purchased from BD Biosciences Pharmingen, and AF700α-CD45R/B220 (clone RA3-6B2), purchased from eBioscience. Plasmablast assays were performed as previously described (59). Activation and differentiation of mouse splenic B cells were induced using a cocktail of mouse IL-4 (1 U/mL; R&D Systems), mouse CD40 ligand (0.6 μg/mL; Stemcell Technologies), and Pam3CSK4 (250 ng/mL; InvivoGen). ES (1 μM) provided pharmacological inhibition of HDAC11. Plasma cell differentiation was assessed by flow cytometry according to changes in CD19, CD138, and B220 surface markers. Data acquired on an LSRII cytometer (Beckman Coulter) were analyzed using FlowJo software (BD Biosciences).

### Viability assays.

For in vitro determination of cytotoxic activity, cells (5 × 10^3^ cells/well) were cultured in 96-well plates in the presence of varying concentrations of indicated agents for specified time periods. Viability was determined using the Cell Counting Kit-8 assay (Dojindo) according to the manufacturer’s instructions.

### Plasmid construction and generation of stably transfected cell lines.

WT (HDAC11^wt^) and enzyme-inactive (HDAC11^mut^) HDAC11 constructs were modified from vectors provided by Ola Witt (German Cancer Research Center; Heidelberg, Germany). The SphI/XbaI fragment of the construct, pEXP3.2-MycHDAC11Mut (48), was subcloned between the SphI/XbaI sites of the HDAC11^wt^ construct, yielding a chimeric construct containing the 5′ end of the WT sequence and the 3′ mutant sequence, generating a mutant construct that has a 5′ sequences (including the Kozak sequence) that is identical to the WT construct. A synthetic sequence for an HA epitope tag was inserted between the PstI site located at the 3′ end of the HDAC11^wt^ and HDAC11^mut^ open reading frames and the XbaI site of the vector, making their 3′ ends identical. Both constructs were subcloned to pRcβactinBleo for stable transfection into myeloma cells. Both constructs were sequenced and were identical to the HDAC11 sequence originally reported by Gao et al. (60). MM1.S cells were transfected with either the HDAC11^wt^.HA or HDAC11^mut^.HA vector or an empty vector using *Trans*IT-LT1transfection reagent from Mirus Bio.

### HDAC11 suppression by siRNA.

siRNA knockdown was accomplished as previously described (61). Briefly, MM1.S cells were seeded in complete medium at a concentration of 2 × 105/mL. After 24 hours, 4 × 106 cells per sample were resuspended in 200 μL Cytomix buffer containing 120 mmol/L KCl, 0.15 mmol/L CaCl_2_, 10 mmol/L K_2_HPO_4_/KH_2_PO_4_, 25 mmol/L HEPES, 2 mmol/L EGTA, 5 mmol/L MgCl_2_, 2 mmol/L ATP, and 5 mmol/L glutathione (pH 7.6), mixed with the indicated ON-TARGET Plus siRNA duplexes targeting HDAC11 (Horizon) at a final concentration of 67 nmol/L, and electroporated at 140 V/975 μF.

### Apoptosis assays.

MM cells were treated as indicated in 6-well plates seeded at a density of 1.0 × 10^6^ cells/well. Treated cells were harvested and processed with Cytofix/Cytoperm buffer before staining with PE-labeled active caspase-3 antibody (PE Active Caspase-3 Apoptosis kit, BD Pharmingen). Viable cells were identified using LIVE/DEAD Fixable Near-IR Dead Cell stain and activated caspase-3–positive cells were gated as an apoptotic population.

### Immunoblotting.

Immunoblotting was performed using previously described methods (62). Equal quantities of protein lysates from different treatments were electrophoresed on SDS-PAGE. Proteins were transferred to a PVDF membrane, blocked with 5% nonfat dry milk or BSA, and incubated with appropriate primary antibodies followed by incubation with secondary antibodies (peroxidase-labeled). Antigen-antibody complexes were visualized by enhanced chemiluminescence.

### qRT-PCR.

Assessment of *HDAC11*, *PRDM1*, *TNFRSF17,* and *GAPDH* expression at the transcriptional level was performed using qRT-PCR as previously described (15). Primer sequences are provided in Supplemental Methods.

### Cell fractionation.

Cytoplasmic fractions were prepared using a cytosolic hypotonic lysis buffer (10 mM HEPES pH 7.9, 10 mM KCl, 0.1 mM EDTA, 0.1 mM EGTA, 1 mM DTT) supplemented with protease/phosphatase inhibitor cocktail. Cell pellets were then resuspended in cold cytosolic lysis buffer and kept on ice for 15 minutes. Then, 10% NP40 was added to the lysis buffer to make the final concentration of 0.075% (v/v) NP40 and vortexed vigorously for 10 seconds, followed by centrifugation at 2,250 x *g* for 5 minutes at 4ºC. The insoluble nuclear pellet was resuspended in a hypertonic nuclear lysis buffer (20 mM HEPES pH 7.9, 400 mM NaCl, 1 mM EDTA, 1 mM EGTA, 1 mM DTT) supplemented with protease/phosphatase inhibitor cocktail and continuously vortexed for 10 seconds every 5 minutes for a total of 30 minutes. After centrifugation at 15,000 x *g* for 10 minutes at 4ºC, the resulting supernatant yielded the nuclear extract.

### Protein IP.

Co-IP was performed using a previously described method (62). After overnight incubation with specific antibodies or normal mouse/rabbit IgG (isotype control), protein A/G PLUS-Agarose beads were added into each sample and further incubated for 3 hours at 4°C with rotation. Immune complexes were pelleted by centrifugation and washed 3 times with IP wash buffer. Finally, beads were resuspended in nonreducing sample buffer and boiled for 5 minutes before loading into SDS-PAGE.

### PLA.

PLA was performed using the Duolink kit (Duolink PLA technology; MilliporeSigma). Fixed cells were incubated with goat anti-IRF4 (sc-11450, Santa Cruz Biotechnology) and mouse anti-HDAC11 (sc-390737, Santa Cruz Biotechnology) primary antibodies, followed by the addition of PLUS (anti-goat) and MINUS (anti-mouse) PLA probes. Ligation and amplification steps were performed following the manufacturer’s protocol. Cells were counterstained with DAPI (nuclei) and α-tubulin–FITC (cytoplasm). Imaging was performed with a Leica SP8 laser scanning confocal microscope through a 63×/1.4NA Plan Apochromat oil immersion objective lens (Leica Microsystems CMS GmbH). Quantitative analysis of PLA signals was performed using the Definiens Enterprise Image Intelligence Suite software.

### Animal experiments.

A syngeneic murine model of MM was established by i.v. inoculation of 5TGM1.*luc* murine myeloma cells into syngeneic C57BL/KaLwRij female mice (Harlan Laboratories; Envigo) of 6–7 weeks of age. Tumor growth was monitored by bioluminescence imaging and serum IgG_2b_ measurement. Mice were randomly distributed into 3 different groups: vehicle control (1% DMSO), ES 1 mg/kg/day, and ES 10 mg/kg/day (*n* = 11/group). Treatment was started 12 days after tumor inoculation. The initial dosing frequency was once daily for the first 10 days. After that, dosing frequency was changed to every alternate day for the rest of the treatment period until mice reached disease endpoint. Drug administration was performed via i.p. injection. Mice were euthanized upon development of hind-limb paralysis, which was considered a surrogate endpoint.

### Ex vivo cytotoxicity assays.

The chemosensitivity of primary MM cells obtained from patients with myeloma was assessed using the Ex vivo Mathematical Malignancy Advisor (EMMA), an ex vivo organoid model system designed to assess drug sensitivity and resistance in patient-derived MM cells in the context of essential bone marrow–derived stromal elements, as previously described (36, 37, 39, 63–65). Briefly, myeloma cells were purified from bone marrow aspirates by CD138 affinity chromatography and plated in a collagen matrix with patient bone marrow stroma and plasma. After incubation overnight, tumor cells were treated with ES, panobinostat, BTZ, or combinations of these drugs and assayed for 96 hours and up to 144 hours using a robotic microscope equipped with incubation chamber (EVOS FL Auto; Thermo Fisher Scientific). The myeloma cell line MM1.S was used in parallel to control for drug potency across experiments. Bright-field images taken every 30 minutes captured cell movement and membrane motion to identify live cells. Mean cell viability of untreated patient-derived MM plasma cells under these conditions was 106.09% ± 17.64% (*n* = 348). Synergy was determined using the method described by Sudalagunta et al. (39), where the percentage of live cells across time and 5 serially diluted (1:3) doses when treated with drugs are used to compute additive response using the Bliss Independence Model. The additive response serves as a reference to determine the extent of synergy observed in each patient sample by comparing it with the percentage of live cells measured when treated with the combination (at a fixed ratio of the 2 constituent single agents). Two metrics of drug sensitivity were employed, AUC and LD_50_, where the additive AUC/LD_50_ was compared with that of the combination to quantify the synergy seen in each patient sample. EMMA provided analyses of cytotoxic activity of individual agents as well as the synergistic potential between HDAC inhibitor and proteasome inhibitor agents.

### ChIP.

Chromatin preparation was performed as previously described (66). Cells (1 × 10^7^ per condition) were lysed in sonication buffer at a density of 3 × 10^6^ cells/130 μL. Lysate was washed twice with sonication buffer and sonicated for 12 minutes in a Covaris ME220 focused-ultrasonicator using AFA microTUBE-130. IRF4 (4964) and normal rabbit IgG (2729) antibodies were obtained from Cell Signaling Technology. Details of qPCR primers for *SUB1*, *TNFRSF17*, and *PRDM1*, identified as targets for IRF4 by Shaffer et al. (24), and myoglobin B used as a control, are provided in Supplemental Methods. Percentage input was calculated by linearization of ΔCt (C_tIP_–C_t1%Input_).

### Proteomics.

Cell lysates were prepared from MM1.S cells conditioned in ES or DMSO for 24 hours. Acetylation of IRF4 was detected and quantified by liquid chromatography tandem mass spectrometry as previously described (67–69). Peptide sequencing and relative quantification were performed on a nanoflow ultrahigh performance liquid chromatograph (Rapid Separation LC; Dionex) interfaced EASY-spray source (Thermo Fisher Scientific) with QE-HFX (benchtop quadrupole-Orbitrap mass spectrometer, Thermo Fisher Scientific). Additional details including quantitation of IRF4 acetylation are provided in Supplemental Methods. Proteome Discoverer (Thermo Fisher Scientific) was used to perform database searches against the UniProt human database using Sequest and Mascot. Skyline (MacCoss Lab software) was used for extracted ion chromatogram quantification.

### Molecular genomics.

DNA extraction was performed on CD138-selected cells isolated from frozen tissues samples using Qiagen QIASymphony DNA purification. RNA extraction was performed using Qiagen RNeasy Plus mini kits. Whole exome sequencing (WES) libraries were prepared using hybrid capture, with an enhanced WES kit (Integrated DNA Technologies), providing double coverage of 440 cancer genes. Library hybridization was performed on an Illumina NovaSeq 6000 instrument. WES was performed on tumor/normal matched samples, generating 100× and 300× coverage, respectively, with 440 cancer genes covered at 600× depth. RNA-Seq was performed using the Illumina TruSeq RNA Exome with single library hybridization, cDNA synthesis, and library preparation and sequencing performed on an Illumina NovaSeq 6000 instrument to 100 million reads per sample. DNA extraction was performed using Qiagen QIASymphony DNA purification. RNA extraction was performed using Qiagen RNeasy Plus mini kits.

Relative gene expression was calculated as *z*-normalized data. Adapter sequences were trimmed from raw tumor sequencing FASTQ file via k-mer matching, quality trimming, contaminant filtering, sequence masking, GC content filtering, length filtering, and entropy filtering. The trimmed FASTQ file was used as the input for the read alignment process using the human genome reference (GRCh38/hg38, available at https://www.ncbi.nlm.nih.gov/assembly/GCF_000001405.39) and the Gencode genome annotation v. 32 using Spliced Transcripts Alignment to a Reference (STAR) software. STAR generated multiple output files used for gene fusion prediction and gene expression analysis. RNA expression values were calculated and reported by RNA-Seq by expectation maximization (RSEM) using estimated mapped reads, fragments per kilobase of transcript per million mapped reads (FPKMs), and transcripts per million mapped reads (TPMs) at the transcript level and gene level based on transcriptome alignment generated by STAR.

Further processing of the RNA-Seq expression data eliminated analytical variability and batch effects associated with preservation methods. Multitier RNA-Seq expression data normalization was performed to generate normalized log_2_(TPM) values. Scaling factors were calculated based on a mean TPM range of 35%–95% for protein-coding genes, and scaled TPM was log_2_ transformed using the following equation: log_2_(TPM + 0.001). Values were LOESS normalized using limma in R (www.r-project.org) with a span of 0.66. To address the normalization of different preservation methods, ComBat (48) was used to eliminate batch effects and the impact of different preservation methods (FBS/DMSO viability preservation, snap frozen, or other methods were used for heme malignancies). Since MM cells were enriched by CD138 positive selection, their transcriptomes were processed separately from other data sets. During this process, outliers were identified by principal component analysis and removed from the data set.

### Data availability.

The molecular and phenotypic data utilized in this manuscript will be made publicly available in Moffitt Cancer Center’s U54 PS-ON/CSBC portal (http://www.dx.doi.org/10.7303/syn25765224). Briefly, the following files will be made publicly available in the U54 PS-ON/CSBC portal: (a) for single sample (ss) gene set enrichment analysis (ssGSEA), files generated by single sample gene set analysis containing normalized enrichment scores, *P* values, and FDRs in the form of.gct files and (b) for GSEA, files generated by GSEA correlating phenotype (ex vivo drug sensitivity to SR3029) and gene expression for 2 gene sets (cancer hallmarks and Kyoto Encyclopedia of Genes and Genomes pathways) will be made available in the form of compressed folders containing the standard file structure generated by GSEA software, including summary of analysis, correlation of individual gene expression and phenotype, and detailed enrichment score for each gene set. Input files for GSEA, expression data set file (.gct), and phenotype (.cis) will be provided in the standard format for GSEA software (http://www.gsea-msigdb.org/gsea/doc/GSEAUserGuideFrame.html?xtools_gsea_Gsea).

### Statistics.

Experiments were performed in triplicate and data are presented as mean ± SD. Statistical comparisons were done in GraphPad Prism (version 7). Mouse data sets were compared using 2-way ANOVA.

### Study approval.

Investigators obtained written informed consent from all patients who were enrolled in Total Cancer Care protocols MCC 14690 and MCC 18608 conducted at the Moffitt Cancer Center & Research Institute, as approved by the IRB. Patient samples were utilized in accordance with the Declaration of Helsinki, International Ethical Guidelines for Biomedical Research Involving Human Subjects (CIOMS), Belmont Report, and US Common Rule. The medical records were deidentified, and only information relating to prebiopsy treatment was reviewed. All mouse studies were conducted strictly in accordance with a protocol reviewed and approved by the IACUC, Research Integrity & Compliance — Research & Innovation at the University of South Florida.

## Author contributions

AGMM designed and performed experiments, coordinated the later stages of the project, and contributed to writing the manuscript. AD designed and performed experiments, coordinated the early stages of the project, and analyzed the results. These authors’ contributions are considered equal; AD was responsible primarily for early concept development and discovery of the functional interaction between HDAC11 and IRF4, and AGMM assumed the lead role in the project upon departure of AD from the lab and was responsible primarily for designing and performing experiments confirming clarifying functional aspects of the HDAC11/IRF4 interaction. MBM reconstructed the plasmids and supervised the ex vivo experiments. ES, JJP, and JPI designed and conducted the eGFP-reported mouse experiment and assessed the plasma cell formation in WT and KO mouse models. AA, GW, and KLW assisted with the design, performance, and analysis of the ChIP-qPCR experiment. AA additionally contributed to performance and analysis of IRF4 functional experiments. AA, DN, RR, and TPN participated in the design and/or interpretation of the reported experiments or results. VI, BF, and JK performed the mass spectrometry experiments to identify the IRF4 acetylation site. GDA helped in patient sample collection. ASS, PS, RRC, and MDCSS designed and planned the ex vivo chemosensitivity assay on primary MM cells. RRA, HAD, and AK helped in clinical data mining. RRA, GDA, OAH, EAW, JKT, and AT helped in clinical data analysis. EMS, KLW, KHS, and WSD provided administrative, technical, or supervisory support. JB and KHS developed the idea for this body of work and supervised the experimental design, scientific analysis of results, and preparation of the manuscript.

## Supplementary Material

Supplemental data

## Figures and Tables

**Table 1:**
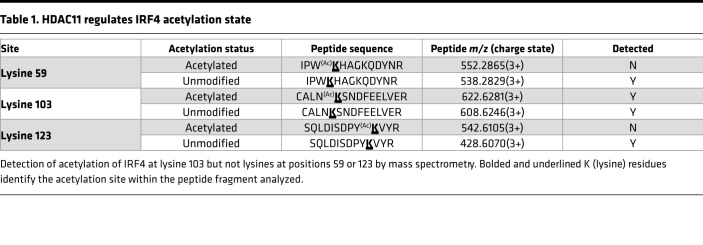
HDAC11 regulates IRF4 acetylation state

## Version 1 11/18/2021

In-Press Preview

## Version 2 12/22/2021

Electronic publication
