# Supplementary material for: Plasma cell dependence on histone/protein deacetylase 11 reveals a therapeutic target in multiple myeloma
Source: JCI Insight. 2021 Dec 22;6(24):e151713. doi: 10.1172/jci.insight.151713 (PMC8783683; doi:10.1172/jci.insight.151713)
Supplement: Supplemental data [file jciinsight-6-151713-s198.pdf]

Supplemental Methods

Quantitation of IRF4 Acetylation by LC-MS with Parallel Reaction Monitoring

After LC-MS/MS analysis of multiple IRF4 digests to identify potential acetylation sites, liquid chromatography-parallel reaction monitoring mass spectrometry (RSLCnano and Q Exactive HF-X, Thermo) was used for targeted quantification of acetylation levels for lysine 103 (K103) using Arg-C digestion, which cleaves the unmodified and acetylated proteins at the same sites to produce peptides that only differ by acetylation (CALNKSNDFEELVER and CALN**AcK**SNDFEELVER, respectively). After immunoprecipitation and recovery by boiling in Laemmli loading buffer, the captured protein was further fractionated by SDS-PAGE. After Coomassie staining (Pierce), the band of interest was excised and destained. After reduction of disulfides, alkylation of cysteines, and in-gel Arg-C proteolytic digestion, peptides were extracted and concentrated by vacuum centrifugation. Stable isotope-labeled standards (Vivitide) were spiked for verification and quantification of the unmodified and acetylated peptides (CALNKSNDFEELVE**R*** and CALN**AcK**SNDFEELVE**R***) as well as the Peptide Retention Time Calibrator mix (Pierce) to assess instrument performance. Peptides were purified by solid phase extraction (SOLAμ, Thermo). LC-PRM data were acquired using reversed phase liquid chromatography (C18 PepMap100, Thermo) with a 35 minute gradient from 2-38.5% B (using the same solvent system described for LC-MS/MS) with an isolation window with of 1.0 m/z with an offset of 0.2 from the monoisotopic m/z value for the peptide, AGC target of 200,000, ion accumulation for up to 500 ms, normalized collision energy 28, and resolution of 30,000. Data were evaluated in Skyline (https://skyline.ms/project/home/software/Skyline/begin.view) and analyzed by calculating light to heavy ratios (for proteolytic peptide from the protein compared to the standard), assessing the amount of the total protein that was acetylated, and finally normalizing the data to the DMSO control.

[illegible]

Primer Sets for qRT-PCR Experiments

Gene	Forward	Reverse
**GAPDH**	5'-GAG TCA ACG GAT TTG GTC GT-3'	5'-TTG ATT TTG GAG GGA TCT CG-3'
**HDAC11**	5'-GAA GTC ACC AGC CCA TAG GT-3'	5'-AGT CAG GAT GGA GGT GGA AG-3'
**PRDM1**	5'-TGA GAG TGC ACA GTG GAG AA-3'	5'-ATT GCT GGT GCT GCT AAA TC-3'
**TNFRSF17**	Purchased from Qiagen, catalog # PPH01178A-200	

Chromosome Immunoprecipitation

Primers for ChIP Experiments

Gene	Forward	Reverse
**SUB1**	5'-GAG TCA ACG GAT TTG GTC GT-3'	5'-TTG ATT TTG GAG GGA TCT CG-3'
**TNFRSF17**	5'-GAA GTC ACC AGC CCA TAG GT-3'	5'-AGT CAG GAT GGA GGT GGA AG-3'
**PRDM1**	5'-TGA GAG TGC ACA GTG GAG AA-3'	5'-ATT GCT GGT GCT GCT AAA TC-3'
**MyoBe2**	Purchased from Diagenode, catalog # C17011006	

Supplemental Figure and Table Legends

Supplemental Figure s1

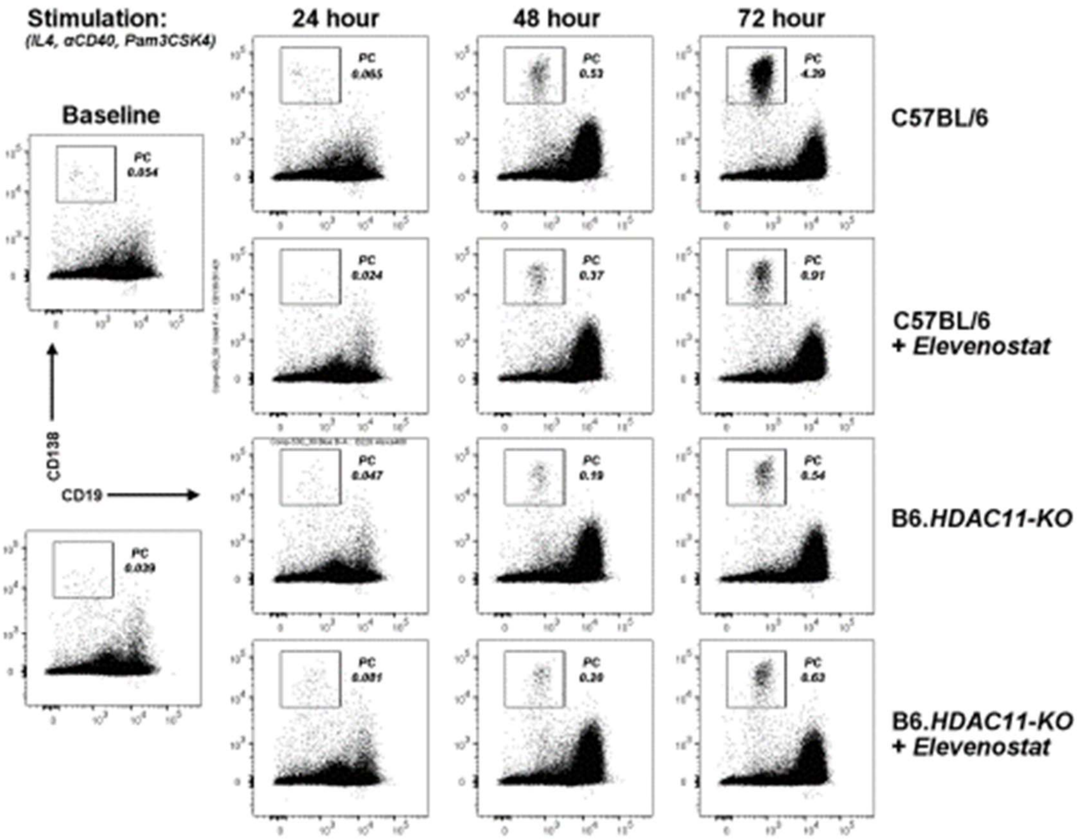

Supplemental Figure s1. Plasma cell differentiation is suppressed in the absence of HDAC11. B cell activation and PC differentiation was induced by stimulation with mouse IL-4 (1 U/ml), mouse CD40 ligand (0.6 µg/ml) along with the TLR agonist, Pam3CSK4 (250 ng/ml). Pharmacological inhibition of HDAC11 was achieved by incubating with elevenostat (1µM). At different time points (24h, 48h, and 72h), cells were collected and processed for flow cytometric analysis to assess the expression of various cell surface markers (CD19, B220, and CD138). Here, PCs were labelled as $CD19^{-}B220^{-}CD138^{+}$.

Supplemental Figure s2

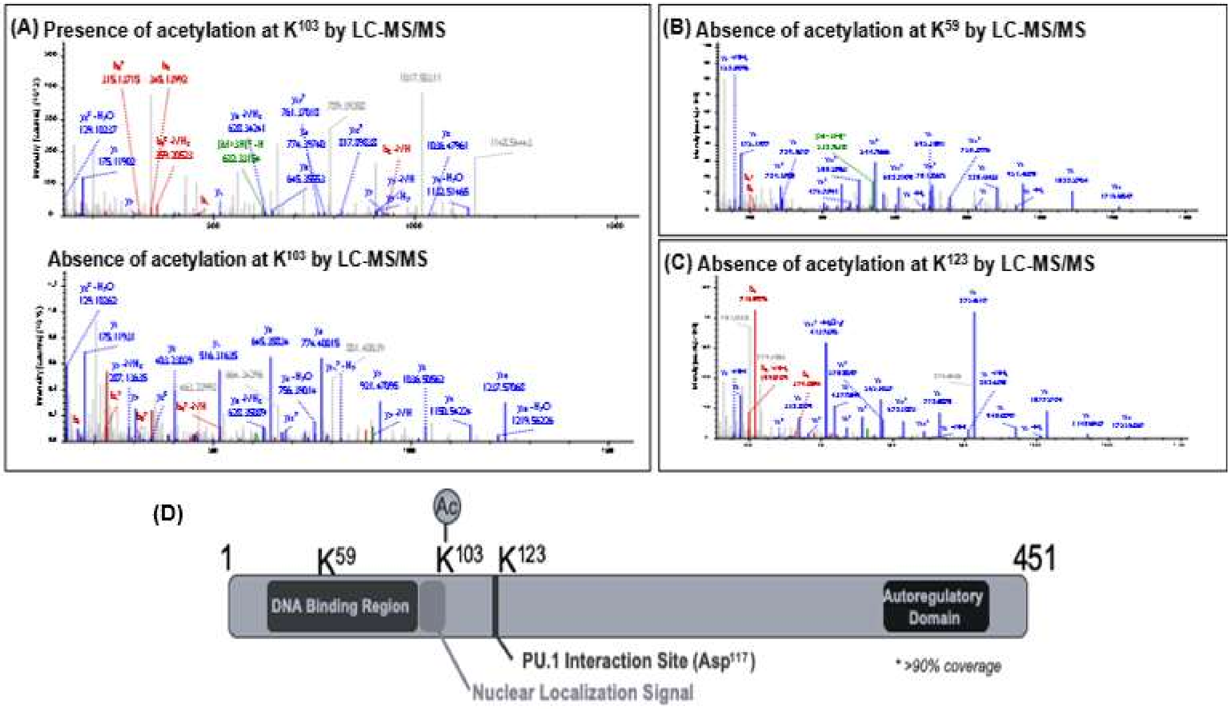

Supplemental Figure s2. Mass spectroscopic analysis indicating acetylation of IRF4 at K^{103}. (A) LC-MS/MS spectrum confirms the sequence CALNKSNDFEELVER with K^{103} being acetylated, detected with m/z: 622.62819 Da (3+), representing a ppm error of 0.11ppm. Unmodified K^{103} was also detected with m/z: 608.62366 Da (3+), representing a ppm error of 1.55ppm. (B) LC-MS/MS spectrum confirms the sequence IPWKHAGKQDYNR with K^{95} being unmodified, detected with m/z: 538.28345 Da (3+), representing a ppm error of 0.93 ppm. (C) LC-MS/MS spectrum confirms the sequence SQLDISDPYKVYR with K^{123} being unmodified, detected with m/z: 608.62366 Da (3+), which represents a ppm error of 1.55ppm. (D) Representation of IRF4 protein depicting lysine amino acid residues proximal to known functional domains, further indicating the acetylation of K103 identified by targeted mass spectrometry.

Supplemental Figure s3.

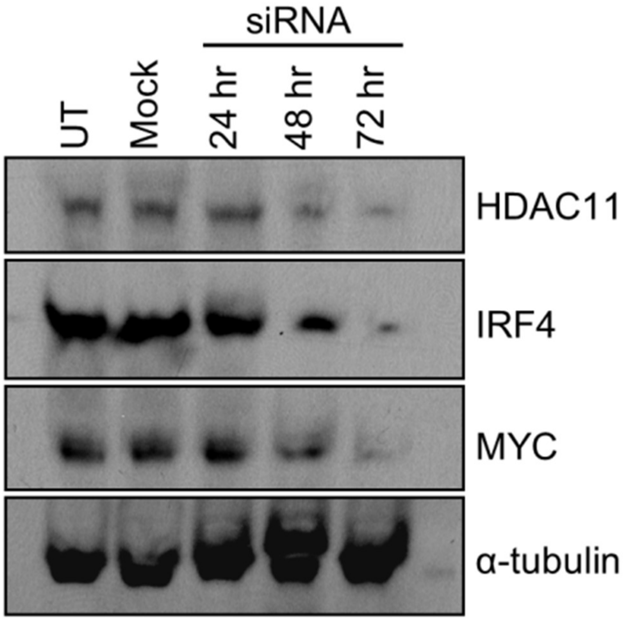

Supplemental Figure s3. HDAC11 knockdown by siRNA. MM1.S cells were depleted of HDAC11 by siRNA. mRNA expression of HDAC11 as well as IRF4 and cMYC was assessed at 24, 48 and 72 hours after treatment with siRNA.

Supplemental Figure s4.

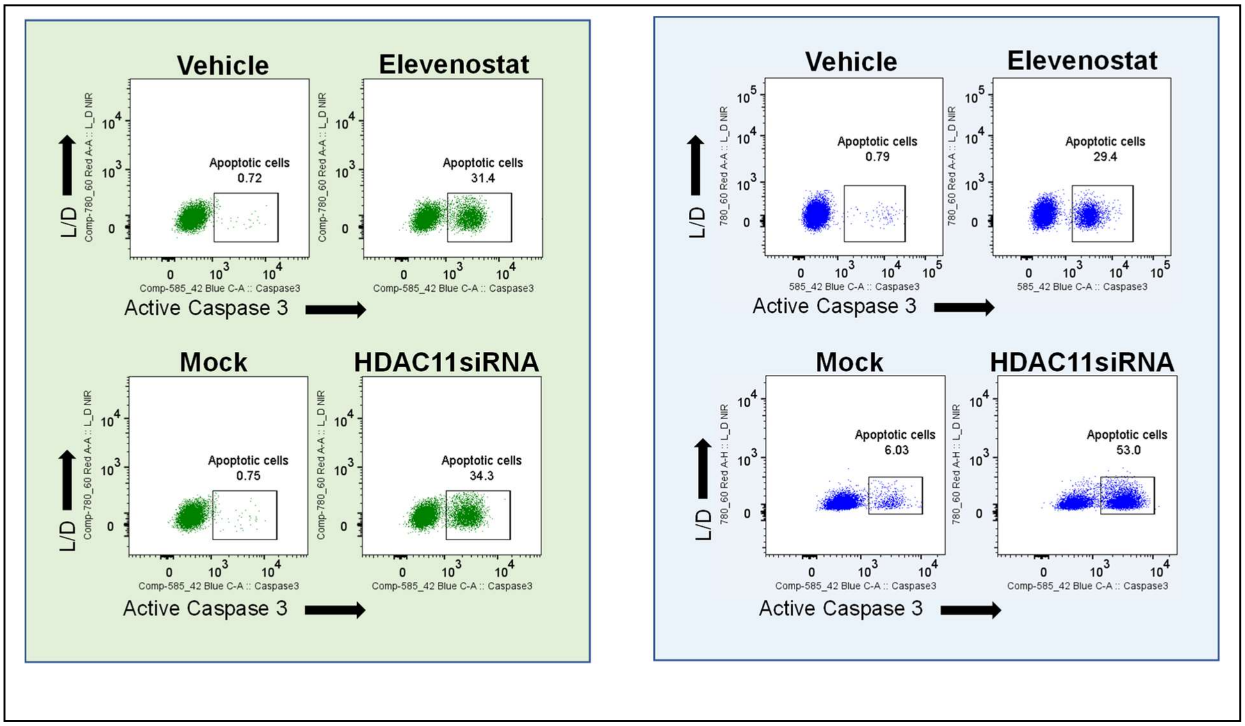

Supplementary Figure s4. HDAC11 inhibition induces caspace-3 activation in MM cells. MM1.S or RPMI-8226 cells were cultured in the presence of ES (IC_{50} dose) or transfected with HDAC11siRNA. Activated caspase-3 was measured by flow cytometry after 48 hours (ES) or 72 hours (HDAC11siRNA). This figure represents results of one iteration of the experiment which was performed 3 times to generate the bar graph included as Figure 3B.

Supplemental Figure s5.

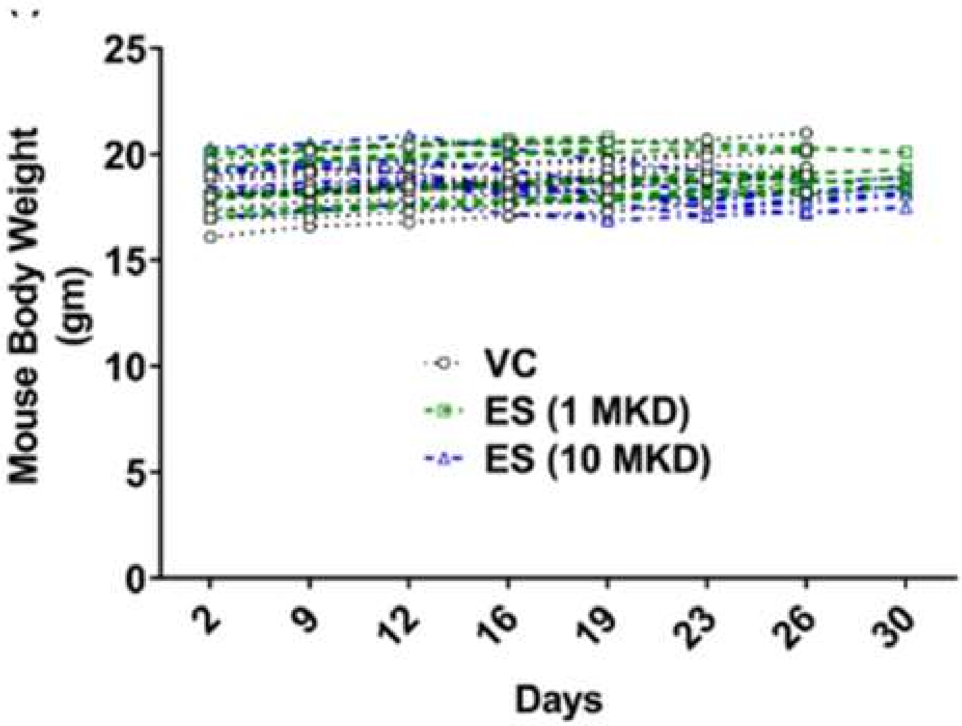

Supplemental Figure s5. Minimal changes in body weight of mice treated with ES suggest low toxicity. C57BL/KaLwRij mice were challenged with 5TGM1-luc murine myeloma cells and subsequently treated ES. Mice were randomly distributed into different groups: vehicle control (VC), elevenostat 1mg/kg/day (ES 1mg/kg/day), and elevenostat 10mg/kg/day (ES 10mg/kg/day) and each group contained 11 mice (n=11). Mouse body weight (gm) measurement was performed twice a week to monitor treatment-induced toxicity.

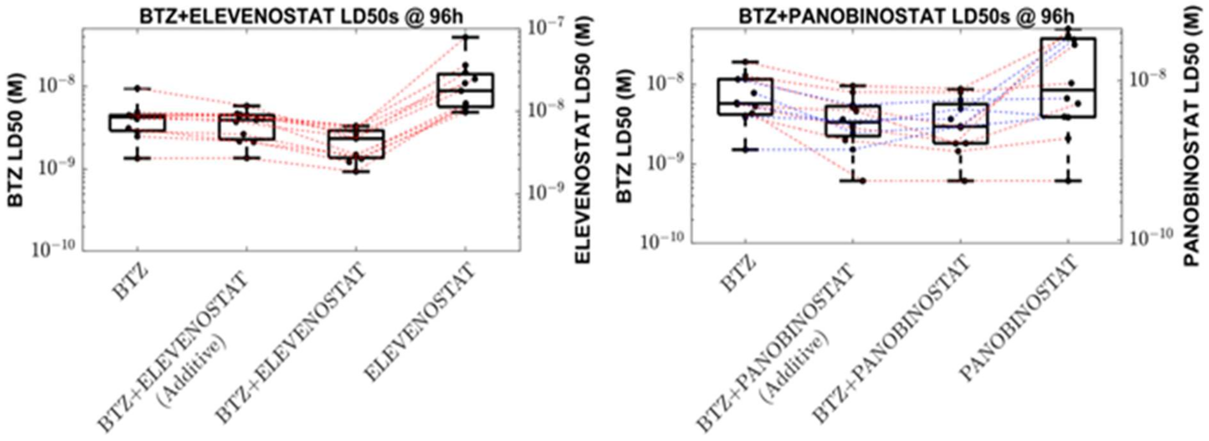

Supplemental Figure s6. Single agent and combination drug sensitivity used to calculate synergy. Lines connect drug response from the same patients, red and blue lines indicate synergy or antagonism, respectively. Outside boxes show single agent activity, center-left boxes show calculated additivity derived from single agent activity, and center-right boxes show actual experimental response from combinations. Mean values and standard deviations are indicated by boxes.
